# Metastatic Small Cell Neuroendocrine Carcinoma of the Prostate: A Rare and Aggressive Case Report From Bangladesh

**DOI:** 10.1155/crom/2821229

**Published:** 2026-09-03

**Authors:** Md. Arifur Rahman, Zinia Jannat Ananna, Mostofa Arafat Islam, Hasin Mesbah Bin Siddique, Mashud Parvez, Sayem Hossain

**Affiliations:** ^1^ Department of Oncology and Radiotherapy, Bangladesh Specialized Hospital PLC, Dhaka, Bangladesh; ^2^ Department of Surgery, Bangladesh Medical University (BMU), Dhaka, Bangladesh; ^3^ Department of Pathology, Bangladesh Specialized Hospital PLC, Dhaka, Bangladesh; ^4^ Department of Urology, Bangladesh Specialized Hospital PLC, Dhaka, Bangladesh

**Keywords:** Bangladesh, FDG PET/CT, immunohistochemistry, neuroendocrine prostate cancer (NEPC), platinum-based chemotherapy, prostate cancer, PSA-discordant prostate cancer, small cell neuroendocrine carcinoma

## Abstract

**Background:**

Small cell neuroendocrine carcinoma of the prostate (SCNEC) is a rare but devastatingly aggressive malignancy, carrying a median survival well under 13 months even with multimodal treatment. It may arise de novo or emerge as a treatment‐resistance phenotype following androgen deprivation in conventional prostatic adenocarcinoma. A defining and clinically treacherous feature is its failure to elevate serum PSA in proportion to tumour burden—a phenomenon termed the ‘PSA paradox’—which routinely delays diagnosis, particularly in resource‐limited settings.

**Case Presentation:**

We report a 35‐year‐old Bangladeshi man who presented with obstructive urinary symptoms, constipation and deep pelvic pain. His serum PSA was only 0.77 ng/mL despite a 6.5 × 6.0 × 5.5 cm prostate mass with liver metastases, extensive osteoblastic bone deposits (SUVmax ~18 on 18F‐FDG PET/CT), left hydronephrosis and regional lymphadenopathy. Initial TRUS‐guided biopsy was reported as Gleason 5 + 5 = 10 adenocarcinoma. Given the PSA–disease burden mismatch, biopsy slides were immediately re‐evaluated; haematuria and acute urinary retention necessitated urgent TURP on 4 December 2024, which confirmed a pure de novo SCNEC with diffuse synaptophysin positivity and a Ki‐67 index exceeding 60%. GATA3 and p40 were negative. Renal function was confirmed adequate before platinum therapy following palliative TURP(serum creatinine 1.2 mg/dL); etoposide–cisplatin (EP) chemotherapy was administered for six cycles with standard hydration, achieving a marked metabolic response on interim PET/CT. Consolidative pelvic IMRT (55 Gy/20 fractions) followed. Despite this approach, the disease relapsed within 3 months; second‐line docetaxel produced no meaningful response. The patient died approximately 11 months from diagnosis.

**Conclusions:**

This case underscores the critical importance of recognising the PSA paradox in younger patients with bulky pelvic disease, the indispensable role of a comprehensive IHC panel in confirming the neuroendocrine phenotype and the urgent need for novel therapeutic strategies in this near‐universally lethal malignancy.

## 1. Introduction

Neuroendocrine carcinoma of the prostate (NEPC), particularly the small cell subtype (SCNEC), accounts for fewer than 1% of primary prostate malignancies at first presentation [1, 2]. A large SEER‐based analysis found that de novo small cell or neuroendocrine carcinoma constitutes only about 0.1% of all incident prostate cancer diagnoses [2]. Far more commonly, neuroendocrine differentiation emerges as an adaptive resistance mechanism following prolonged androgen deprivation—occurring in an estimated 10%–25% of men with metastatic castration‐resistant prostate cancer (treatment‐related NEPC, t‐NEPC) [3]. The median age at NEPC diagnosis hovers between 68 and 72 years [4], making our patient′s presentation at 35 years—with fully metastatic, de novo SCNEC—an extreme clinical outlier.

Clinically, SCNEC nearly always declares itself late. Visceral metastases (liver and lung) and extensive bone involvement are typical at diagnosis [3, 5], yet the tumour produces little or no PSA—the so‐called ‘PSA paradox’ [6]. Confirming the diagnosis demands histopathological vigilance and a thorough immunohistochemical panel, because a subset of SCNEC (~10%–15%) fails to express any of the three classic neuroendocrine markers (synaptophysin, Chromogranin A and CD56), leaving morphology as the primary diagnostic anchor [4, 7]. Once confirmed, prompt platinum‐based chemotherapy—largely extrapolated from small cell lung cancer (SCLC) protocols given the absence of SCNEC‐specific trials—forms the cornerstone of management [8–10].

## 2. Clinical Presentation

**Figure 1:**
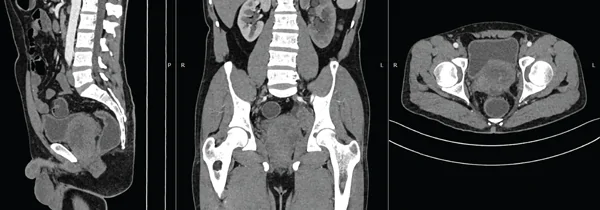
Contrast‐enhanced CT (CECT) of the abdomen and pelvis at diagnosis (November 2024), in sagittal, coronal and axial planes. A grossly enlarged, lobulated prostate mass (6.5 × 6.0 × 5.5 cm) causes bladder outlet obstruction and left hydronephrosis. Enlarged pelvic lymph nodes are present.

Figure 1 CECT of the abdomen and pelvis at diagnosis (November 2024).

A 35‐year‐old man with no prior medical history presented to the Department of Oncology and Radiotherapy, Bangladesh Specialized Hospital (BSH) PLC, Dhaka, in late 2024 with a 6‐week history of progressive obstructive urinary difficulties, burning micturition, chronic constipation and deep‐seated anal pain. On examination he was ambulant and self‐caring, with an ECOG performance status of 1 at presentation. Systemic examination was otherwise unremarkable, with no palpable abdominal mass, organomegaly or peripheral lymphadenopathy . Digital rectal examination revealed a markedly enlarged, hard, nodular prostate. Serum PSA was just 0.77 ng/mL (reference < 4.0 ng/mL)—a value so discordant with his symptoms that it immediately raised the suspicion of a PSA‐invisible high‐grade malignancy [6]. Serum neuron‐specific enolase (NSE) and Chromogranin A were not measured at presentation, an acknowledged limitation in a resource‐constrained context. There was no family history of prostate, breast, ovarian, pancreatic or colorectal malignancy and no known hereditary cancer syndrome in first‐ or second‐degree relatives . He was a lifelong non‐smoker with no significant alcohol intake and no occupational chemical exposure . He was married, employed and independent in all activities of daily living before presentation ].

The urgent urethrocystoscopy, clot evacuation, and palliative TURP performed on 4 December 2024 decompressed the bladder outlet. The bleeding source at the bladder neck was identified and controlled. A 22 Fr three‐way Foley catheter was left in situ, and tissue was sent for histopathology. Left hydronephrosis had been identified on imaging secondary to ureteral compression by the prostate mass. Renal function was assessed before cisplatin‐based chemotherapy: serum creatinine was 1.2 mg/dL with an eGFR of 84 mL/min/1.73 m² immediately before the first EP cycle on 14 December 2024. Cisplatin was administered with standard pre‐ and post‐hydration (at least 1–2 litres of normal saline before and after each dose) throughout the six‐cycle course.

## 3. Imaging

Transrectal ultrasound (TRUS) confirmed markedly heterogeneous prostatic enlargement. Contrast‐enhanced CT (CECT) of the abdomen and pelvis demonstrated a lobulated prostate mass measuring 6.5 × 6.0 × 5.5 cm, exerting mass effect on the bladder base and causing left ureteric obstruction with hydronephrosis. Multiple enlarged pelvic and inguinal lymph nodes (1–1.5 cm) were identified, and hypodense lesions in both hepatic lobes were consistent with metastatic deposits (Figure 1).

Whole‐body 18F‐FDG PET/CT on 23 November 2024 revealed an intensely hypermetabolic disease burden extending well beyond the pelvis. The prostate demonstrated heterogeneous FDG uptake (SUVmax ~8.5) with central necrosis; numerous pelvic lymph nodes along the internal iliac and perirectal chains were FDG‐avid (SUVmax ~6.5). Scattered lesions throughout the axial and appendicular skeleton (SUVmax up to ~18) corresponded to extensive osteoblastic bone metastases, and several hepatic foci were FDG‐avid (SUVmax 3.7–6.5). The glaring discordance between this metabolic burden and the normal PSA essentially confirmed a nonadenocarcinomatous phenotype from the outset.

## 4. Histopathology and Immunohistochemistry

### 4.1. Biopsy and Initial Diagnosis

The initial TRUS‐guided prostate biopsy (December 2024) was reported as a poorly differentiated prostatic adenocarcinoma, Gleason 5 + 5 = 10 (Grade Group 5), with focal perineural invasion but no definitive lymphovascular invasion. The PSA–disease burden mismatch led the treating team to request immediate rereview of the biopsy slides and additional IHC studies. While this process was underway, the patient developed acute urinary retention and haematuria secondary to the bulky prostate mass, necessitating an urgent TURP on 4 December 2024 for urinary decompression, haemostasis and additional tissue acquisition.

### 4.2. TURP Histopathology

**Figure 2:**
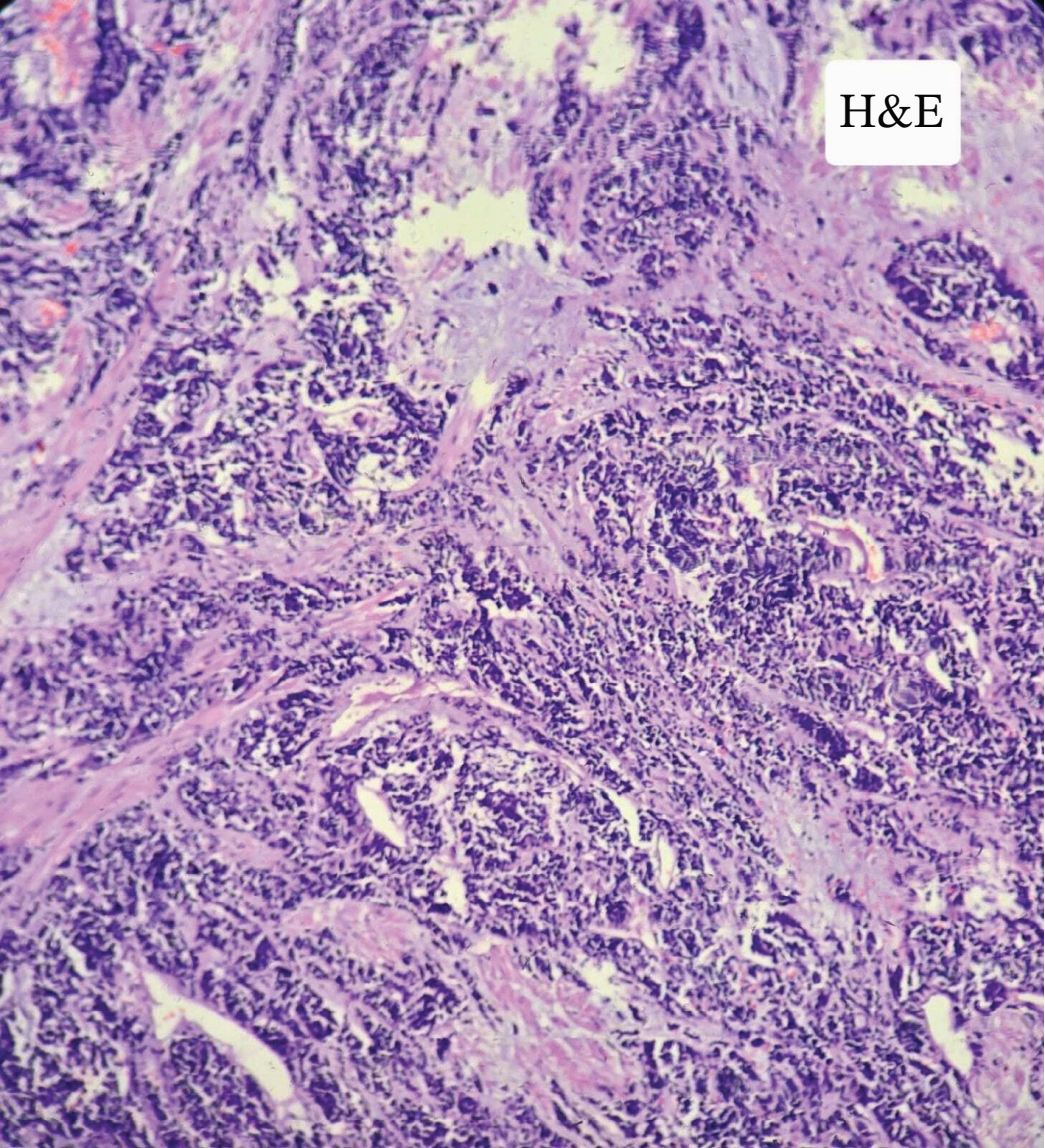
Haematoxylin and eosin (H&E)–stained section of the TURP specimen. Diffuse sheets of malignant small cells with hyperchromatic, moulded nuclei and scant cytoplasm, areas of coagulative necrosis and a high nuclear‐to‐cytoplasmic ratio. Crush artefact is present.

Histological examination of the TURP chips revealed a pure small cell neuroendocrine carcinoma. The tumour exhibited the archetypal ‘small blue cell’ morphology: diffuse sheets and nests of small round‐to‐oval malignant cells with exceedingly scant cytoplasm, finely granular (‘salt‐and‐pepper’) chromatin, frequent nuclear moulding and a high mitotic count. Geographic coagulative necrosis and a desmoplastic stromal reaction were both present. No acinar (glandular) architecture was identified on H&E staining, strongly supporting a primary de novo neuroendocrine lineage rather than adenocarcinoma with neuroendocrine differentiation (Figure 2). Concurrent rereview of the initial biopsy corroborated this interpretation—the original Gleason 5 + 5 diagnosis reflected morphological overlap between poorly differentiated adenocarcinoma and SCNEC on limited biopsy tissue rather than genuine mixed histology.

### 4.3. Immunohistochemical Profile

**Figure 3:**
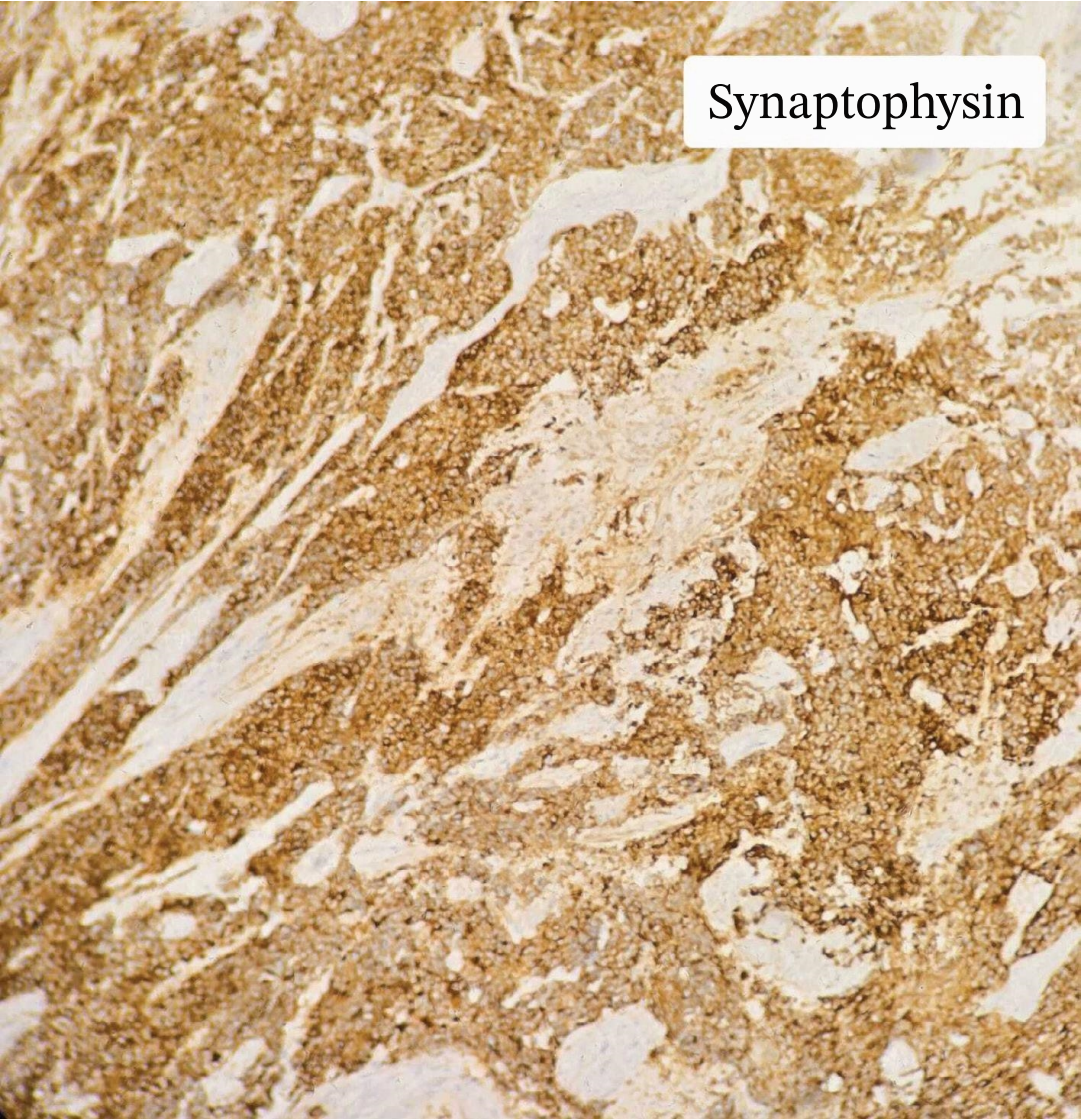
Synaptophysin immunohistochemistry. Diffuse, strong cytoplasmic positivity in virtually all tumour cells, confirming neuroendocrine lineage.

**Figure 4:**
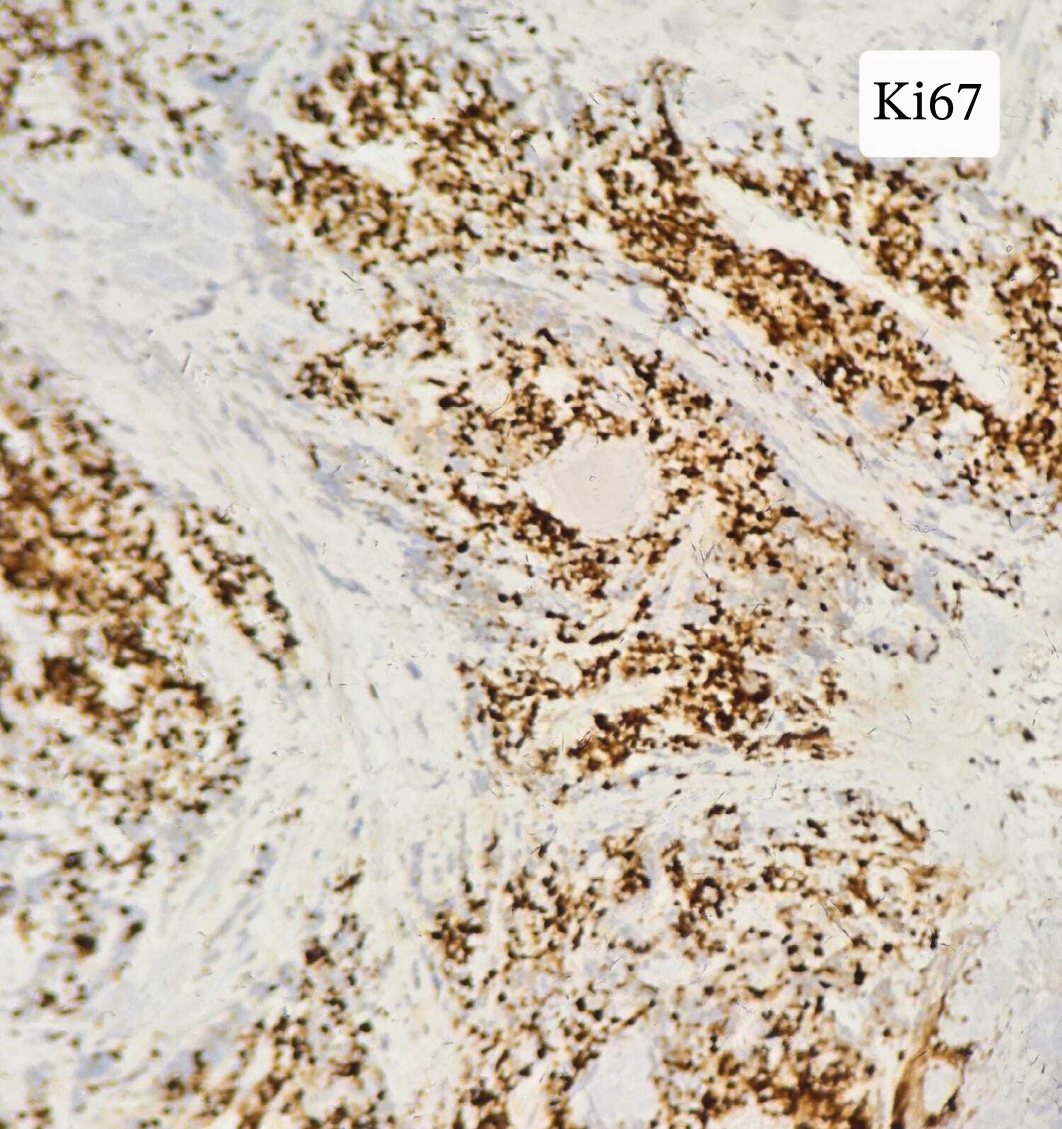
Ki‐67 immunostain. Labelling index exceeds 60% of tumour cell nuclei.

**Figure 5:**
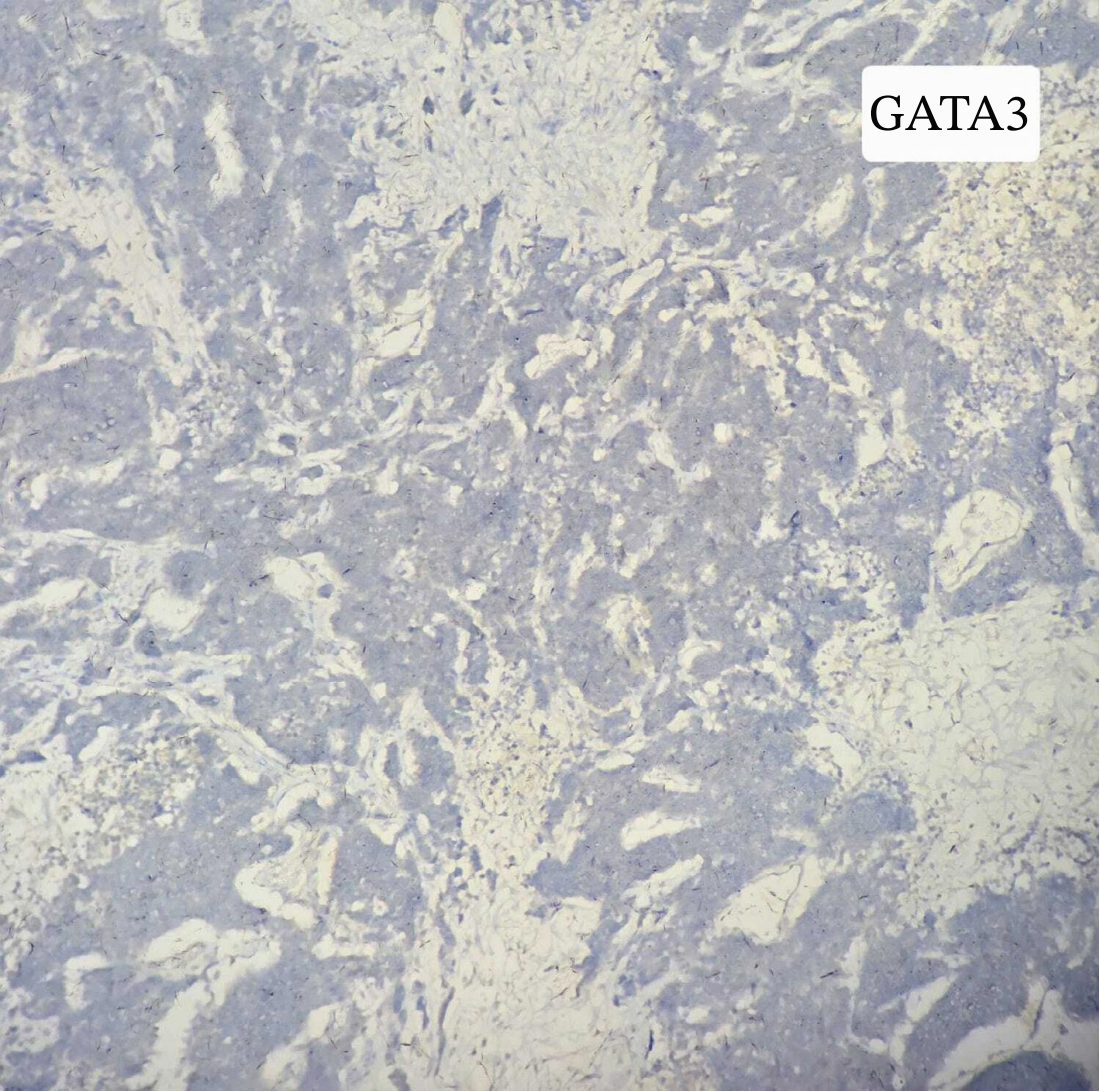
GATA3 immunostain (negative). Complete absence of nuclear staining effectively excludes a urothelial primary.

**Figure 6:**
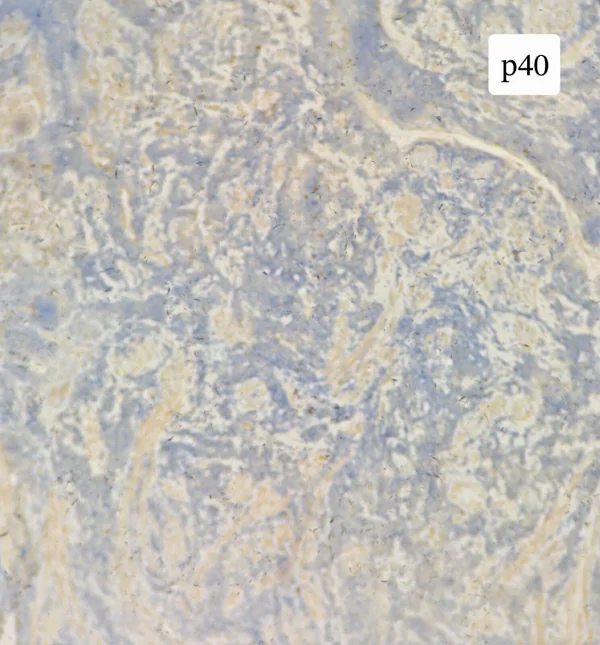
p40 (*Δ*Np63) immunostain (negative). Absence of staining argues against a squamous cell carcinoma component, consistent with a pure neuroendocrine phenotype.

Immunohistochemically, the tumour showed diffuse, strong cytoplasmic synaptophysin positivity (Figure 3) in virtually all tumour cells, confirming a neuroendocrine lineage. Ki‐67 labelling index exceeded 60% (Figure 4), in keeping with the highly proliferative nature of SCNEC. GATA3 was completely negative (Figure 5), ruling out urothelial carcinoma; p40 (*Δ*Np63 isoform) was also negative (Figure 6), effectively excluding a squamous cell component. Chromogranin A and CD56 staining were not available in our resource‐limited setting at the time of diagnosis—an acknowledged limitation of this case, discussed further below. Similarly, TTF‐1, INSM1, NKX3.1, PSA and PSAP immunostains were not performed; however, the clinical context (bulky prostatic primary, extensive local infiltration and absent lung lesions on imaging) made metastatic pulmonary small cell carcinoma effectively excluded on clinicoradiological grounds. Androgen receptor (AR) immunostaining was also not performed, another acknowledged limitation.

Figure 2 H&E section showing small blue cell morphology.

Figure 3 Synaptophysin immunohistochemistry (diffuse positive).

Figure 4 Ki‐67 immunostain (> 60% index).

Figure 5 GATA3 immunostain (negative).

Figure 6 p40 immunostain (negative).

The integrated pathological and clinicoradiological findings established the diagnosis of metastatic small cell neuroendocrine carcinoma of the prostate, clinical stage T4 N1 M1c (AJCC 8th edition: M1b for osseous disease, M1c for visceral metastases; M1c assigned as the highest category applies), with no admixed conventional adenocarcinoma component.

## 5. Treatment Course

### 5.1. Surgical Debulking

The first intervention was the therapeutic TURP on 4 December 2024, which not only obtained diagnostic tissue but also decompressed the urinary tract and controlled haematuria. The multidisciplinary team—urologists, medical oncologist and radiation oncologist—convened and agreed on a systemic therapy–first strategy. The complete chronology of the patient′s clinical course, investigations and interventions is summarised in Table 1.

### 5.2. First‐Line Chemotherapy

Etoposide (100 mg/m^2^/day) plus cisplatin (25 mg/m^2^/day), each given on Days 1–3 of a 21‐day cycle (EP), the standard first‐line regimen for extensive‐stage small cell carcinoma per NCCN and analogous SCLC guidelines, was commenced on 14 December 2024 [8, 10]. Prechemotherapy renal function had been confirmed as adequate, and cisplatin was administered throughout with standard pre‐ and posthydration. The patient completed six cycles at 3‐weekly intervals, finishing on 5 April 2025. Tolerability was acceptable; moderate fatigue and haematological toxicity were managed conservatively without dose reductions. Concurrent monthly zoledronic acid was initiated for bone‐protective effect.

### 5.3. Response to Chemotherapy

**Figure 7:**
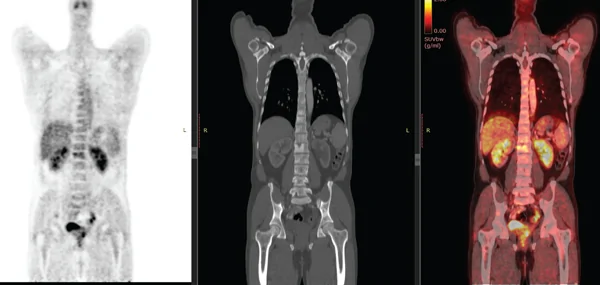
18F‐FDG PET/CT after six cycles of etoposide–cisplatin (May 2025). Prostatic and nodal FDG uptake is markedly reduced compared with baseline. Previously hypermetabolic hepatic and osseous lesions show near‐complete metabolic resolution, indicating a significant partial remission.

Restaging 18F‐FDG PET/CT on 1 May 2025 documented a significant partial metabolic remission. Prostatic SUVmax fell from ~8.5 to ~5.2; many previously hypermetabolic bone deposits had become sclerotic and metabolically quiescent, consistent with treatment effect; hepatic metastases showed near‐complete resolution of FDG activity. A few low‐SUV residual pelvic nodes persisted (Figure 7). Serum PSA had risen modestly to ~2.2 ng/mL—unsurprising, as PSA is a poor response biomarker in SCNEC.

Figure 7 FDG PET/CT postchemotherapy (May 2025) showing significant partial metabolic remission.

### 5.4. Consolidative Therapy

In view of the favourable response and residual pelvic disease, consolidative intensity‐modulated radiotherapy (IMRT) was delivered to the prostate and pelvic lymph nodes (55 Gy in 20 fractions) between late May and early July 2025. No established radiotherapy dose‐fractionation schedule exists for SCNEC; this prescription was adapted from hypofractionated protocols used in high‐risk adenocarcinoma [10] and was intended primarily for local disease control and symptom palliation rather than cure. Treatment was completed without interruption and produced modest improvement in lower urinary tract symptoms.

### 5.5. Adjunct and Maintenance Therapy

Goserelin 3.6 mg 4‐weekly (ADT) was added in July 2025 as an adjunct, primarily to suppress any possible occult adenocarcinomatous component given the initial biopsy misclassification, even though pure SCNEC characteristically lacks AR‐driven growth and derives minimal benefit from hormonal therapy [5, 6]. AR immunostaining was not performed—an acknowledged limitation—so AR status remained unknown; this was therefore a pragmatic rather than evidence‐based decision. Maintenance oral etoposide 50 mg once daily (21 days on, 7 days off cycles) was continued after EP completion, extrapolated from SCLC practice [8], given the absence of validated maintenance strategies in prostatic SCNEC; no guideline endorses this approach in this setting. The patient remained on surveillance through mid‐2025.

### 5.6. Disease Progression

By August 2025—barely 3 months after achieving best response—the patient developed new‐onset severe lower back and right‐sided chest pain. Repeat imaging confirmed disease relapse with new osteoblastic metastases in the lumbar spine and ribs. Palliative radiotherapy was delivered to symptomatic skeletal lesions: 30 Gy in 10 fractions to an L1 vertebral metastasis and a single 8 Gy fraction to a painful right rib lesion. These provided partial but meaningful pain relief.

### 5.7. Second‐Line Therapy

**Figure 8:**
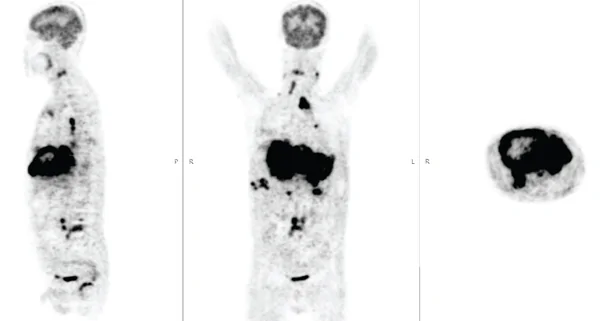
18F‐FDG PET/CT at disease progression (September 2025). Maximum intensity projection images show widespread metabolically active disease with multiple new FDG‐avid lesions in the axial and appendicular skeleton and re‐emergent hepatic uptake, reflecting rapid relapse characteristic of SCNEC.

By September 2025, follow‐up FDG PET/CT showed widespread relapse with numerous new osseous and nodal lesions and re‐emergence of hepatic FDG uptake (Figure 8). Brain imaging revealed no intracranial involvement. Topotecan—the established second‐line agent in relapsed SCLC—was unavailable; temozolomide‐based regimens and lurbinectedin were also inaccessible in our setting. Weekly low‐dose docetaxel (30 mg/m2 iv) [11] was commenced on 21 September 2025—a tolerability‐focused regimen chosen in recognition of his ECOG performance status of 3 at that point. As is typical for refractory SCNEC, the tumour was essentially unresponsive and the disease progressed inexorably over the following weeks.

Figure 8 FDG PET/CT at disease progression (September 2025).

## 6. Outcome

The patient′s condition deteriorated rapidly through October 2025, shortly after the second weekly dose of docetaxel. He was admitted for management of intractable pain, profound weakness and disease‐related functional decline. Despite maximal supportive and palliative care, he died of progressive disease in late October 2025—approximately 11 months from initial diagnosis. No post‐mortem examination was performed, but the clinical trajectory was entirely consistent with overwhelming metastatic disease burden. The full timeline from symptom onset to death is presented in Table 1.

## 7. Timeline

**Table 1:** Chronological summary of the patient′s clinical course, investigations and interventions from symptom onset to death.

Date	Clinical events, investigations and interventions
Mid‐October 2024	Onset of progressive obstructive urinary symptoms, burning micturition, chronic constipation and deep pelvic pain.
November 2024	DRE: markedly enlarged, hard, nodular prostate. Serum PSA 0.77 ng/mL. TRUS and CECT: prostate mass 6.5 × 6.0 × 5.5 cm, bilobar hepatic lesions, left hydronephrosis, pelvic/inguinal lymphadenopathy.
23 November 2024	Whole‐body 18F‐FDG PET/CT: prostate SUVmax ~8.5 with central necrosis; pelvic nodes SUVmax ~6.5; axial and appendicular skeletal lesions SUVmax up to ~18; hepatic foci SUVmax 3.7–6.5.
Early December 2024	TRUS‐guided biopsy reported as poorly differentiated adenocarcinoma, Gleason 5 + 5 = 10 (Grade Group 5). PSA–burden mismatch prompts slide rereview and additional IHC.
4 December 2024	Acute urinary retention with haematuria. Urgent urethrocystoscopy, clot evacuation and palliative TURP. Tissue sent for histopathology.
5 December 2024	Serum creatinine 1.3 mg/dL; PSA 2.0 ng/mL; alkaline phosphatase 78 U/L.
8–12 December 2024	Haemoglobin 10.7–11.0 g/dL; packed cell transfusion given. Serum sodium 136 mmol/L. Creatinine 1.2 mg/dL.
December 2024	TURP histopathology: pure small cell neuroendocrine carcinoma. Synaptophysin diffusely positive; Ki‐67 > 60%; GATA3 negative; p40 negative. Stage T4 N1 M1c.
14 December 2024	Cycle 1 of etoposide (100 mg/m^2^/day) plus cisplatin (25 mg/m^2^/day), Days 1–3, 21‐day cycle. Monthly zoledronic acid commenced.
10 February 2025	PSA 2.25 ng/mL; creatinine 1.0 mg/dL. Treatment continuing without dose reduction.
5 April 2025	Sixth and final cycle of EP completed. Tolerability acceptable; moderate fatigue and haematological toxicity managed conservatively.
1 May 2025	Restaging 18F‐FDG PET/CT: significant partial metabolic remission. Prostate SUVmax 8.5 → 5.2; osseous lesions sclerotic and metabolically quiescent; near‐complete hepatic response.
Late May to early July 2025	Consolidative IMRT to prostate and pelvic nodes, 55 Gy in 20 fractions. Completed without interruption; modest improvement in urinary symptoms.
July 2025	Goserelin 3.6 mg 4‐weekly commenced. Maintenance oral etoposide 50 mg once daily (21 days on, 7 days off).
August 2025	New severe lower back and right‐sided chest pain. Imaging confirms relapse with new lumbar and rib metastases. Palliative RT: 30 Gy/10 fractions to L1; single 8 Gy fraction to right rib.
18–26 September 2025	Widespread relapse on FDG PET/CT with new osseous, nodal and hepatic disease. No brain metastases. Sodium 126–127 mmol/L; haemoglobin 10.3–11.3 g/dL.
21 September 2025	Second‐line weekly docetaxel 30 mg iv commenced (ECOG 3).
3–7 October 2025	Sodium 123–127 mmol/L; ALT 160–178 U/L; bilirubin 1.4–1.8 mg/dL; leucocytosis (WBC 31 × 10^9^/L) with atypical cells on film. No response to docetaxel.
18 October 2025	Creatinine 1.9 mg/dL; sodium 130 mmol/L; potassium 5.4 mmol/L; albumin 31 g/L. Admission for intractable pain and functional decline.
Late October 2025	Death from progressive disease, approximately 11 months after initial diagnosis. No post‐mortem examination performed.

## 8. Patient Perspective

The patient was not able to provide a written perspective on his illness and treatment. He remained under active oncological care until his terminal admission and died in late October 2025, and no formal patient‐perspective statement had been recorded before his death. His family, who provided consent for this publication, expressed that early recognition of the diagnosis and clear communication about the aggressive nature of the disease had been the aspects of care they valued most.

## 9. Consent for Publication

### 9.1. No Written Consent, No Identifiable Information

No written consent has been obtained from the patient as there is no patient‐identifiable information included in this case report. All clinical data, laboratory results and images presented have been fully de‐identified.

## 10. Discussion

### 10.1. Diagnostic Lessons From This Case

The most instructive aspect of this case is the diagnostic trajectory itself. A 35‐year‐old man—some 3–4 decades below the published NEPC median of 68–72 years [4]—presented with bulky, multiorgan metastatic disease and a PSA of 0.77 ng/mL. Had PSA and Gleason score been treated as the sole arbiters of risk, as they frequently are in resource‐limited settings, the neuroendocrine nature of the tumour might have been fatally delayed in its recognition. Instead, the mismatch between the clinical picture and the biochemical marker became the diagnostic pivot point. The PSA paradox—a recognised hallmark of SCNEC in which minimal PSA elevation coexists with overwhelming tumour burden [6]—should trigger active consideration of a neuroendocrine phenotype whenever it is observed, irrespective of patient age.

The initial biopsy misclassification as Gleason 5 + 5 = 10 adenocarcinoma also merits reflection. Small biopsy cores from poorly differentiated SCNEC can be morphologically indistinguishable from high‐grade adenocarcinoma on routine H&E alone, particularly when neuroendocrine markers are suboptimally expressed [4]. Approximately 10%–15% of SCNECs lack the expression of all common neuroendocrine markers on standard immunohistochemistry [4, 7], underscoring that morphological assessment by an experienced uropathologist—not reflex reliance on a single IHC panel—remains the diagnostic gold standard. In our case, the PSA paradox served as the tripwire that triggered biopsy rereview; the TURP specimen then provided abundant tissue for a definitive diagnosis.

We acknowledge several IHC limitations. Chromogranin A, CD56 and INSM1—three markers that together with synaptophysin constitute the WHO 2022–recommended panel for SCNEC [1]—were not performed, primarily due to resource constraints. TTF‐1, NKX3.1, PSA and PSAP immunostains were similarly unavailable. These gaps are genuine diagnostic limitations, mitigated by the strong clinicoradiological evidence for a prostatic primary. We document them transparently so that clinicians in similarly resource‐limited settings can prioritise the most discriminating markers—synaptophysin, Ki‐67, GATA3 and p40—when the full panel is unattainable. Molecular profiling was also not performed; in an ideal setting, SCNEC typically harbours biallelic TP53 and RB1 loss, MYCN and AURKA amplifications and DLL3 overexpression [12–14]. Given the patient′s young age, germline testing for BRCA1/2, mismatch repair deficiency and Lynch syndrome should also have been considered [12]—a call to develop genomic infrastructure in lower‐income healthcare systems.

### 10.2. Functional Imaging in Prostatic SCNEC

Conventional prostate cancer imaging tools—bone scintigraphy, CT and even 68Ga‐PSMA PET/CT—frequently underestimate disease extent in SCNEC. Neuroendocrine tumours often downregulate both androgen receptor signalling and PSMA expression, rendering PSMA‐targeted tracers relatively insensitive [3, 15]; interpretation is further complicated by the known normal variants and artefacts of PSMA PET/CT [16]. Poorly differentiated SCNEC is instead characterised by markedly elevated glycolytic metabolism, producing avid FDG uptake that accurately reflects tumour burden even when PSA is normal [15]. In our patient, 18F‐FDG PET/CT was selected from the outset as the staging modality of choice. The baseline scan mapped multiorgan disease that had completely eluded laboratory markers; the postchemotherapy scan then provided the objective metabolic response data that guided the consolidative radiotherapy decision.

68Ga‐DOTATATE PET/CT—which identifies somatostatin receptor (SSTR)–expressing lesions and can stratify patients for peptide receptor radionuclide therapy (PRRT) with 177Lu‐DOTATATE [17]—was not performed in this case, primarily because of resource unavailability. SSTR expression in poorly differentiated SCNEC is variable and often low or absent [17], so the clinical yield is uncertain; nevertheless, the absence of DOTATATE imaging meant that PRRT candidacy could not be assessed—a genuine missed opportunity, particularly relevant after platinum failure. In resource‐adequate settings, DOTATATE PET/CT alongside FDG PET at baseline in SCNEC patients would be recommended, as dual‐tracer imaging can fully characterise disease heterogeneity [5].

### 10.3. Clinical Course and Response to Treatment

Our patient′s disease trajectory closely mirrors the natural history of metastatic SCNEC documented in the literature. Median overall survival ranges from approximately 7 months in treatment‐emergent cases to around 12 months in de novo disease, with a 5‐year overall survival rate of roughly 8% [2, 3, 5, 6]. His 11‐month survival, despite aggressive multimodal therapy, sits squarely within this grim range—a salutary reminder that even a young, previously healthy individual is not spared the aggressive trajectory of this tumour.

The initial EP chemotherapy response was gratifying: PET/CT demonstrated near‐complete hepatic metabolic remission and widespread osseous sclerosis. Yet durability was brief. Disease progression occurred approximately 3 months after best response, precisely in line with reported median PFS of 3–4 months following first‐line EP in SCNEC [11]. Once platinum resistance had developed, second‐line docetaxel produced no meaningful response—consistent with the general lack of effective salvage options in this setting [11]. This patient′s experience reinforces the consensus: Once SCNEC escapes platinum, there is currently no reliable systemic salvage.

Local therapies played a meaningful palliative role throughout. The urgent TURP addressed urinary obstruction and haematuria while simultaneously securing the diagnostic tissue [4, 9]. Targeted radiotherapy to symptomatic bone lesions improved pain control and functional status when systemic options were exhausted; the value of directed radiotherapy to dominant lesions in neuroendocrine prostate cancer, albeit reported in the ablative rather than the palliative setting, has been described previously [18]. These modalities, although not altering the ultimate disease trajectory, significantly improved quality of life over the clinical course.

### 10.4. Therapeutic Strategy and Emerging Directions

The management of SCNEC is, by necessity, extrapolated from SCLC protocols—a position made uncomfortable by the biological differences between the two entities [8–10]. Cisplatin (or carboplatin) combined with etoposide remains the undisputed first‐line backbone; in prospective research, a subset of clinically defined ‘anaplastic’ variant castration‐resistant prostate cancers has been shown to respond favourably to platinum‐based regimens [19], lending supporting evidence for this approach across neuroendocrine‐spectrum disease. Androgen signalling inhibitors (abiraterone and enzalutamide) are generally ineffectual in pure SCNEC due to the characteristic absence of AR‐driven growth [5, 6] and were not administered here beyond the pragmatic goserelin adjunct.

Several novel therapeutic strategies are under investigation for NEPC, though none were applied or available to this patient. DLL3 overexpression, identified as a recurrent feature of NEPC in genomic series [12], has prompted the development of DLL3‐targeted antibody–drug conjugates [13]. PARP inhibitors may benefit the subset of NEPC patients harbouring homologous recombination repair defects [13]. PRRT with 177Lu‐DOTATATE represents a theranostic avenue for SSTR‐expressing lesions [17], and Aurora kinase A inhibition (alisertib) has shown occasional responses in a Phase II trial [20]. These remain investigational: None constitutes current standard of care. They are mentioned here to contextualise the unmet need and to highlight the importance of molecular profiling at diagnosis and enrolment in clinical trials wherever feasible.

## 11. Conclusion

De novo small cell neuroendocrine carcinoma of the prostate is rare, lethal and diagnostically treacherous—particularly when the treating clinician operates in a setting where PSA and Gleason grade function as the primary risk‐stratification tools. This case documents the full clinical arc of the disease in an unusually young patient: from the PSA paradox that initially misdirected diagnosis, through urgent TURP and IHC confirmation, to an initial EP response followed by rapid relapse and death within 11 months. Several acknowledged limitations—incomplete IHC panel, absence of molecular profiling and no DOTATATE imaging—reflect real‐world constraints in resource‐limited settings and are documented here precisely so that clinicians in comparable environments can plan prospectively to close these gaps.

The educational message is clear: In any patient presenting with bulky pelvic disease and a PSA that seems paradoxically reassuring, neuroendocrine differentiation must enter the differential without delay. A comprehensive IHC panel, FDG PET/CT staging, formal assessment of renal function before platinum chemotherapy and early MDT involvement are the non‐negotiable pillars of optimal management. Durable remission remains out of reach with current therapies; only collaborative research, expanded access to molecular diagnostics and inclusion of NEPC patients in clinical trials offer any realistic hope of meaningfully altering this disease′s trajectory.

## Funding

No funding was received for this manuscript.

## Ethics Statement

This case report was prepared and reported in accordance with the CARE (CAse REport) guidelines.

## Conflicts of Interest

The authors declare no conflicts of interest.

## Data Availability

The data that support the findings of this study are available on request from the corresponding author. The data are not publicly available due to privacy or ethical restrictions.
